# Robot-Assisted Minimally Invasive Direct Coronary Artery Bypass Graft in High-Risk Patients with Reduced Left Ventricular Function: Comparison with Conventional Off-Pump Coronary Artery Bypass Graft Surgery

**DOI:** 10.3390/medicina62081519

**Published:** 2026-08-06

**Authors:** Laura Besola, Iside Folcarelli, Federico Giorgi, Matteo Mazzola, Danilo Ruggiero, Andrea Colli

**Affiliations:** 1 Cardiac Surgery Unit, Department of Surgical, Medical and Molecular Pathology and Critical Care, University of Pisa, Via Paradisa 2, 56124 Pisa, Italy; laura.besola@hotmail.it (L.B.);; 2 Cath Lab Unit, Department of Surgical, Medical and Molecular Pathology and Critical Care, University of Pisa, Via Paradisa 2, 56124 Pisa, Italy

**Keywords:** RA-MIDCAB, off-pump CABG, minimally invasive coronary revascularization, restricted mean survival time

## Abstract

*Objective*: Robotic-assisted minimally invasive direct coronary artery bypass (RA-MIDCAB) is a proven alternative to conventional off-pump sternotomy for revascularizing the left anterior descending artery (LAD) in low-risk patients. However, its role in higher-risk candidates remains poorly established. This study compares RA-MIDCAB with off-pump CABG (OPCAB) in intermediate/high-risk patients with moderately reduced left ventricular ejection fraction (LVEF). *Methods*: We retrospectively analyzed all consecutive high/intermediate-risk patients with depressed LVEF who underwent left internal mammary artery to LAD RA-MIDCAB or OPCAB at our center between January 2021 and August 2025. The primary outcome, assessed at discharge and follow-up, was a composite of all-cause mortality, stroke, and repeat revascularization. Secondary outcomes included ICU stay, red blood cells (RBC) transfusion volume, oro-tracheal intubation time, and acute kidney injury. Kaplan–Meier curves estimated the cumulative freedom from the primary outcome. Restricted mean survival time (RMST) at 1 year was calculated, with non-inferiority confirmed if the lower bound of the 95% CI exceeded − 8 days. A generalized linear model adjusted for confounders. *Results*: Seventy-two patients were included: 20 RA-MIDCAB and 52 OPCAB. EuroSCORE was 3.8 ± 1.9 and 3.5 ± 2.4 in RA-MIDCAB and OPCAB, respectively (*p* = 0.384), while mean LVEF was 40 ± 13 and 45 ± 9, respectively (*p* = 0.454). Other variables were comparable; however, RA-MIDCAB patients had higher rates of prior myocardial infarction (MI) (45% vs. 11.5%; *p* = 0.003) and PCI (40% vs. 13.4%; *p* = 0.022). Procedural time was longer for RA-MIDCAB (196 ± 66 vs. 182 ± 66 min; *p* = 0.048). Thirty-day mortality was comparable (0% RA-MIDCAB, 3.8% OPCAB; *p* = 0.374), while ICU stay and intubation time were shorter in the RA-MIDCAB group. Fewer RA-MIDCAB patients required >5 RBC units. At one year, freedom from the primary outcome was 95% vs. 83.6% (*p* = 0.2), with RMST favoring RA-MIDCAB by 34.6 days (*p* = 0.0067), meeting the non-inferiority threshold. Results remained consistent after adjustment. *Conclusions*: RA-MIDCAB is noninferior to OPCAB in intermediate- to high-risk patients with moderately depressed LVEF, offering a valuable alternative to conventional surgical revascularization. These findings need further confirm through the analysis of larger datasets and longer follow-up.

## 1. Background

Coronary artery disease (CAD) is one of the major causes of death and disability worldwide, particularly in Western countries [1]. Coronary Artery Bypass Grafting (CABG), either on-pump or off-pump (OPCAB), has proven to be superior to optimal medical therapy and percutaneous revascularization (PCI) in the presence of left main (LM) disease, multivessel disease (MVD), chronic total occlusion (CTO), high coronary complexity, and diabetes [2,3,4,5,6,7]. In particular, the use of the left internal mammary artery (LIMA) to revascularize the left anterior descending artery (LAD) has been shown to increase survival and long-term graft patency [8]. Whilst it provides optimal long-term outcomes, it is still affected by the long recovery time associated with sternotomy. Indeed, long-term follow-up (FU) studies, such as the STICH and STICHES trials, demonstrated that surgical myocardial revascularization in high-risk patients reduces mortality by approximately 16% compared with optimal medical therapy (OMT) [9,10].

In recent years, minimally invasive techniques, including Minimally Invasive Direct Coronary Artery Bypass (MIDCAB) and robotic assisted minimally invasive direct coronary artery bypass (RA-MIDCAB), have emerged with the purpose of reducing perioperative risk related to full sternotomy and to cardiopulmonary bypass (CPB), such as inflammation, neurological events, kidney injury, and the necessity of blood transfusions [11,12]. In properly selected patients, RA-MIDCAB also provides lower infection rates, reduced intensive care unit (ICU) and hospital length of stay, and fewer red blood cell transfusions [13]. The advent of less invasive techniques in patients for whom conventional strategies have an increased perioperative risk and who would alternatively undergo percutaneous coronary intervention (PCI) represents an important alternative [14,15], in particular in patients with LAD CTO or isolated LAD stenosis. With respect to PCI, studies demonstrated that RA-MIDCAB has superior long-term outcomes compared with PCI, including MACE, new myocardial infarction, and the need for repeat revascularization [16]. RA-MIDCAB, alone or combined with PCI to complete the revascularization (hybrid coronary revascularization, HCR), has been demonstrated to be equally safe as conventional off-pump CABG, also in patients with left ventricular dysfunction [17]. Since its diffusion is limited by a steep learning curve [18,19], the literature comparing RA-MIDCAB to conventional CABG is still relatively limited, especially in high-risk populations.

The aim of this study is to assess the early results of RA-MIDCAB in high/intermediate-risk patients with reduced left ventricular ejection fraction (LVEF), comparing them to conventional OPCAB.

## 2. Methods

This retrospective observational study includes all consecutive patients with a LVEF of ≤50% and/or a high/intermediate risk score according to the calculated EuroSCORE and STS Score (>3%) who underwent revascularization with LIMA to LAD via RA-MIDCAB or full-sternotomy OPCAB at our institution between January 2021 and August 2025. In our institution, RA-MIDCAB was introduced in September 2023; however, we extended the inclusion period to January 2021 for statistical reasons. Patients in the robotic group could have undergone isolated LIMA-to-LAD revascularization, hybrid revascularization (surgery followed by PCI), or inverse hybrid revascularization (PCI followed by surgery). Patients who underwent the hybrid strategy usually completed the revascularization during the same hospitalization. Differently, those who underwent the inverse hybrid technique received PCI for an acute coronary syndrome and, depending on the severity of residual coronary lesions and clinical features, could have completed revascularization either during the same hospitalization or after 30 days during new access. All patients signed a written informed consent; the study was conducted in accordance with the Declaration of Helsinki and approved by the local Ethics Committee.

### 2.1. RA-MIDCAB Procedure

RA-MIDCAB was performed using a standard technique in a conventional operating room (OR). All procedures were performed using the Da Vinci Xi platform (Surgical Intuitive, Sunnyvale, CA, USA). The patient is under general anesthesia with oro-tracheal selective intubation and intraoperative transesophageal echocardiography monitoring. With the patient in 30° right lateral decubitus and the left arm lowered to flatten the left shoulder, after exclusion of the left arm and induction of CO2 pneumothorax, three robotic trocars are placed in the 2nd (black diamond or micro bipolar forceps), 4th (30° camera) and 6th (hook or spatula) intercostal spaces, between the mid-clavicular line and the anterior axillary line. The robot is docked, and the LIMA is then identified and harvested in a skeletonized method. The pericardium is opened, and the optimal site for the anastomosis is selected. After LIMA harvesting, through a mini-thoracotomy (4–5 cm), the surgeon performs the LIMA-LAD anastomosis with the beating heart, avoiding extracorporeal circulation and cardioplegia.

Baseline, intraoperative, and postoperative parameters were collected. Clinical visit and transthoracic echocardiography (TTE) were performed preoperatively, at discharge, and during follow-up, usually at 1–3 months and yearly thereafter, at the discretion of the local cardiologist. Relative data were obtained via telephone interview or chart review.

### 2.2. Outcomes

The study’s primary outcome, evaluated at discharge and last follow-up, was a combined outcome of all-cause death, myocardial infarction (MI) (new ST-segment elevation/depression in more than three consecutive leads and/or new negative T-waves, modification of postoperative troponin [>10 times the 99th percentile URL in patients with normal baseline troponin values. In patients with elevated preprocedure troponin in whom troponin levels are stable (≤20% variation) or falling, the postprocedure troponin must rise by >20%] and/or new myocardial wall motion abnormalities on echocardiography who underwent an angiography study for these reasons), stroke (assessed with CT scan and/or neurological clinical evaluation), and necessity of repeat revascularization. Secondary endpoints were also evaluated, including hospital and 1-year mortality, length of stay in ICU, number of transfused RBC units, oro-tracheal (OT) intubation time, and acute kidney injury (increase in postoperative creatinine within 72 h of surgery, 150% with respect to baseline).

### 2.3. Statistical Analysis

Continuous variables are presented as mean ± standard deviation or median (interquartile range), depending on data distribution, and were compared using Student’s t-test or Mann–Whitney U test, as appropriate. Categorical variables are reported as counts and percentages and were compared using the chi-square test or Fisher’s exact test. Kaplan–Meier (KM) survival curves were generated to estimate cumulative freedom-from-event rates, and differences between groups were assessed using the log-rank test. To provide a more robust, clinically interpretable measure, the restricted mean survival time (RMST) at 1 year was calculated. RMST reflects the average survival time within a fixed follow-up horizon, does not rely on the proportional hazards assumption, and—by incorporating the entire survival curve—is less sensitive to the absolute number of observed events compared with conventional hazard ratio analysis. The between-group difference in RMST was expressed with 95% confidence intervals, and non-inferiority was concluded if the lower bound of the interval was greater than the prespecified non-inferiority margin (−8 days), which was previously used also in other cardiovascular trials [20,21]. No formal test for superiority was planned. In addition, a generalized linear model with binomial distribution and logit link was used to estimate adjusted event probabilities for each group. Adjusted risk differences with 95% confidence intervals were derived from these estimates. To account for potential confounding while maintaining model parsimony, covariates—age, sex, previous MI, and previous PCI—were entered separately into the models. For this analysis, non-inferiority was concluded if the upper bound of the 95% confidence interval for the adjusted risk difference was below the prespecified non-inferiority margin on the absolute risk scale (+8 percentage points). A two-sided significance level of α < 0.05 was considered statistically significant. All analyses were performed using SPSS version 28 (Spss Inc., Chicago, IL, USA) and MedCalc 20.1.10 (MedCalc Software Ltd.).

## 3. Results

Seventy-two patients were included; 20 underwent RA-MIDCAB and 52 OPCAB. Mean age was 73 ± 9 and 69 ± 10 years in the RA-MIDCAB and OPCAB groups, respectively (*p* = 0.092). The two populations did not differ for baseline characteristics and cardiovascular risk factors as shown in Table 1. Surgical risk scores were similar in the two groups: EuroSCORE was 3.8 ± 1.9% and 3.5 ± 2.4%, respectively, in the RA-MIDCAB and OPCAB groups (*p* = 0.384), while the STS Score was 3 ± 2% and 2.4 ± 2%, respectively (*p* = 0.577). Most patients were symptomatic with an NHYA functional class III (95% RA-MIDCAB, 94.2% OPCAB) and experienced similar rates of previous hospitalization for heart failure (5% RA-MIDCAB and 7.7% OPCAB). All patients were treated with guideline-directed medical therapy, and only one patient from both groups was on dual antiplatelet therapy (DAPT). Patients in the RA-MIDCAB group more frequently experienced a previous myocardial infarction (MI) than those in the OPCAB group (45% vs. 11.5%, *p* = 0.003) and underwent more previous PCI (40% vs. 13.4%, *p* = 0.022). On preoperative TTE, LVEF was 40 ± 13% and 45 ± 9% in the RA-MIDCAB and OPCAB groups, respectively (*p* = 0.454). Approximately 30% of patients in both populations had moderate/severe mitral valve regurgitation (MR), and moderate pulmonary hypertension was seen in 10% of RA-MIDCAB patients and 1.9% of OPCAB patients (*p* = 0.194). Complete baseline TTE data are available in Table 2.

All patients underwent single arterial revascularization for the LAD. Complete revascularization was achieved in all RA-MIDCAB patients and in 92% (48) of OPCAB. Skin-to-skin time was longer in the robotic group compared with conventional surgery (196 ± 66 vs. 182 ± 66 min, *p* = 0.048). During the procedure, inotropic/vasopressor support was required in 20% of cases in the RA-MIDCAB group and in 12% of cases in the OPCAB group (*p* = 0.352), and one patient in the OPCAB group required an Intra-Aortic Balloon Pump.

Extubation within 6 h from the OR was possible in all RA-MIDCAB patients (80% extubated in the OR) and in 54% of OPCAB patients (*p* = 0.001). Similarly, ICU stay tended to be shorter in the RA-MIDCAB group compared with the OPACB group (ICU stay > 24 h: 35% vs. 31%, respectively; *p* = 0.05). Thirty-day overall mortality was 4% (2 patients) in the OPCABG group while no deaths were observed in the RA-MIDCAB group (*p* = 0.374). Peak Troponin within 72 h of surgery was lower in the RA-MIDCAB group (133.4 ± 92.2 vs. 524.6 ± 93.4 mcg/L, *p* = 0.014). One patient in the OPCAB group developed perioperative MI and underwent coronary angiography and PCI on the previously by-passed vessel for LIMA failure. New onset postoperative atrial fibrillation (POAF) was observed in 25% of RA-MIDCAB patients and 31% in the off-pump group (*p* = 0.06). We observed two cases of AKI in the off-pump group but none in the RA-MIDCAB group. We had one wound dehiscence and three cases of sepsis in the OPCAB group but these differences had no statistical significance. Transfusion with more than one pool of RBC was necessary in 11 (21%) patients in the OPCAB group and one patient (5%) in the RA-MIDCAB group (*p* = 0.099). The total length of stay was longer in the OPCAB group with respect to the RA-MIDCAB group (6.4 ± 1.5 and 6 ± 1.9 days, *p* = 0.029). Transfer to a rehabilitation facility was necessary in 8 (40%) patients in the RA-MIDCAB group and in 35 (70%) patients in the OPCAB group (*p* = 0.033). At discharge, LVEF improved in all patients. Complete perioperative results are reported in Table 3.

**Figure 1:**
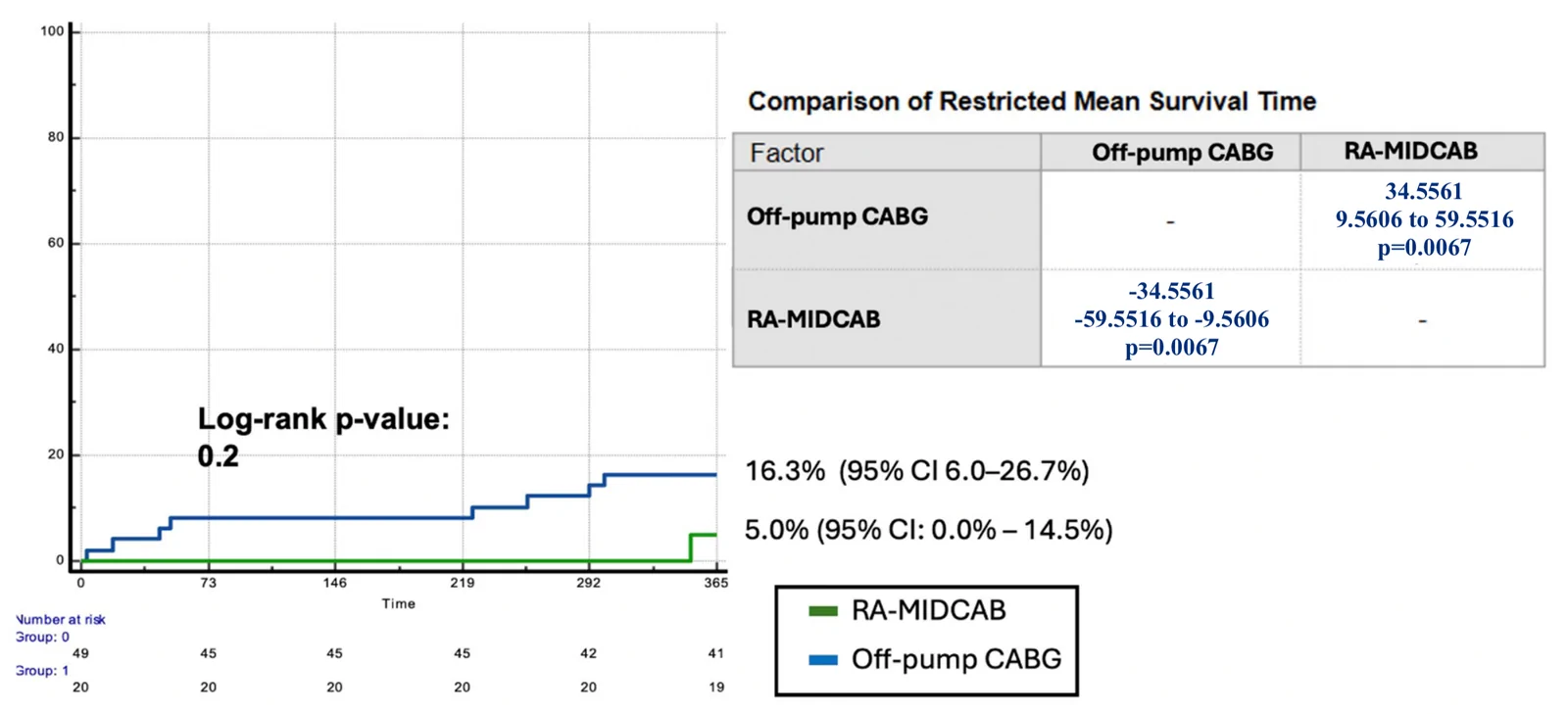
Kaplan–Meier curve showing freedom from primary outcome in the two groups and restricted mean survival time analysis in the table on the right.

**Figure 2:**
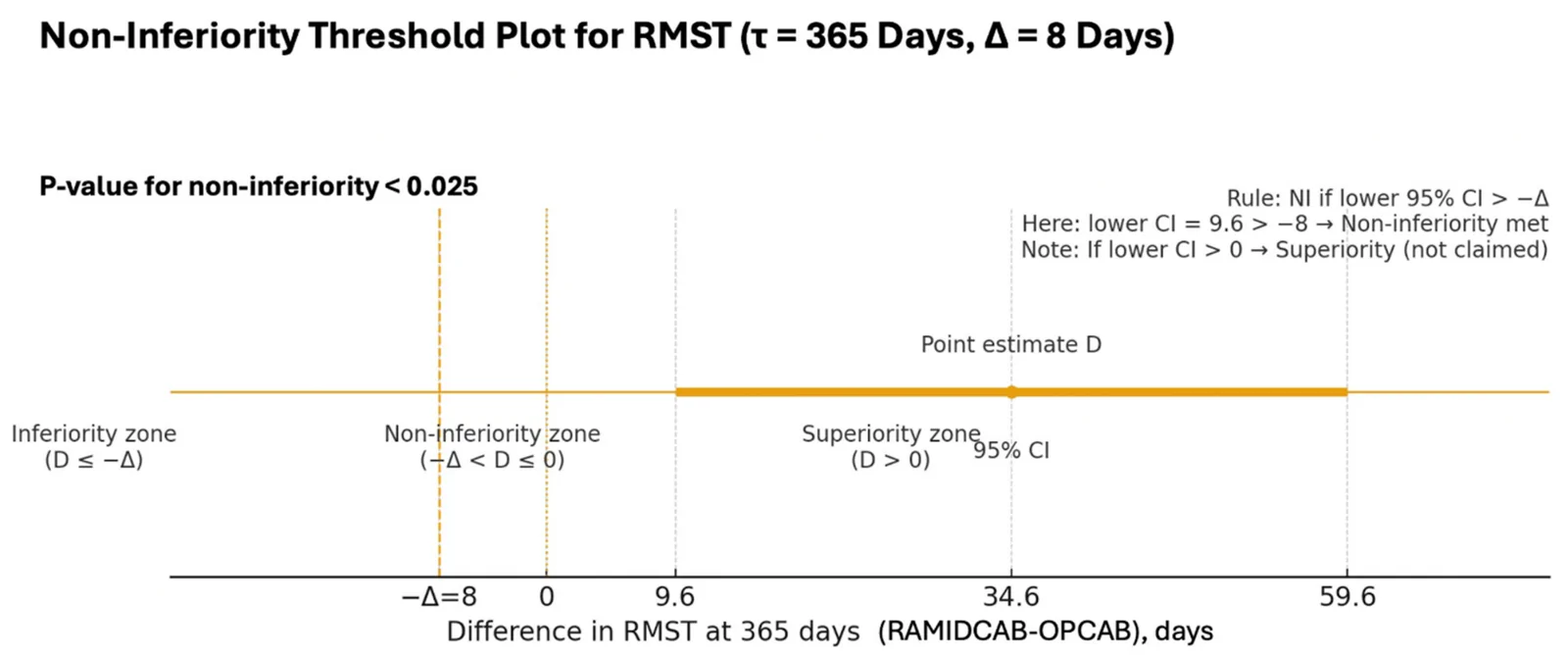
Non-inferiority analysis. The pre-specified non-inferiority margin was Δ = 8 days. Since the lower bound of the 95% CI (+9.6 days) is greater than −Δ, non- inferiority of the RAMIDCAB procedure is demonstrated. No formal test for superiority was planned; therefore, no superiority claim is made.

Median follow-up time for the entire population was 456 days (I-III quartiles 218.5–771.3 days), 232 days (54.5–537 days) for the RA-MIDCAB and 519 days (288.8–893.3 days) for the OPCAB groups. No patients were lost at follow-up and follow-up completeness was 100% for clinical data and 83.5% for echocardiographic data. At 1-year follow-up, mortality was 5% and 18% (*p* = 0.068) in the RA-MIDCAB and OPCABG groups, respectively. At last FU LVEF was 50 ± 10.4% and 50 ± 11 in the two groups, respectively (*p* = 0.749), with a mild tendency to better improvement in the OPCAB group than in the RA-MIDCAB group (∆LVEF 5% and 8%, respectively, *p* = 0.071). The KM analysis showed a cumulative incidence of the primary outcome of 16.3% (95% CI: 6.0–26.7%) in OPCAB patients and 5% (95% CI: 0–14.5%) in RA-MIDCAB patients (*p* = 0.2). At one year, the difference in RMST (RA-MIDCAB vs. OPCAB) was 34.6 days in favor of RA-MIDCAB (*p* = 0.0067, 95% CI 9.6–59.6) (Figure 1). The pre-specified non-inferiority margin was Δ = 8 days. The lower bound of the 95% CI was 9.6 days, greater than the preset, demonstrating the non-inferiority of the RA-MIDCAB procedure. No formal test for superiority was planned; therefore, no superiority claim could be made. (Figure 2). Non-inferiority was maintained also after adjustment for confounding variables as age (1-year risk difference −10%, 95% CI −24–5%, *p* = 0.007), sex (1-year risk difference −10%, 95% CI −24–4%, *p* = 0.006), prior MI (1-year risk difference −10%, 95% CI −27–7%, *p* = 0.019), and prior PCI (1-year risk difference −10%, 95% CI −27–7%, *p* = 0.014).

## 4. Discussion

Minimally invasive revascularization techniques, as MIDCAB, RA-MIDCAB, and Total Endoscopic Coronary Artery Bypass (TECAB), have proven to be safe and effective in patients with CAD, offering shorter recovery times and improved quality of life compared to conventional full-sternotomy CABG [22,23,24,25,26]. Most of the published studies have assessed the effects of RA-MIDCAB in low-risk patients, while a few included high-risk patients with depressed LVEF [27]. Our study adds preliminary knowledge regarding intermediate/high-risk patients with moderately to severely reduced cardiac function. The main findings of our study are:

(1) Patients at intermediate/high-risk patients with moderately reduced LVEF who underwent RA-MIDCAB experienced 34.6-day longer survival with respect to OPCAB which is enough to confirm the non-inferiority of the procedure but not to confirm superiority.

(2) With respect to OPCAB, RA-MIDCAB tends to provide shorter OT intubation time, ICU and total length of stay time.

(3) Postoperative complications were similar between groups, RA-MIDCAB tended to provide a lower incidence of POAF and lower requirement of RBC transfusion but with borderline statistical significance.

While at one year the cumulative occurrence of the primary outcome was similar between the two groups, the RMST analysis showed that patients who underwent RA-MIDCAB seemed to experience a slightly longer freedom from events with respect to OPCAB, mainly driven by a lower mortality suggesting the procedure is not inferior to standard sternotomy approach in more complex patients. To our knowledge this is the first study assessing the survival advantage in patients treated with RA-MIDCAB. Previous studies showed that this technique was associated with a comparable survival rate and a repeat revascularization rate compared with conventional off-pump revascularization [28,29]. Su et al. reported better in-hospital and 20-year follow-up outcomes in patients undergoing multivessel RA-MIDCAB compared with OPCAB, suggesting a protective effect of RA-MIDCAB. While the study presented a larger population, the two populations showed significant preoperative disparities, possibly impacting results. In our study, the two groups are mostly comparable, thus limiting confounding. In the study by Algoet et al. [19], there was no difference in postoperative major events or 3-year survival between two matched populations who underwent RA-MIDCAB or OPCAB for MVD. In our report, RA-MIDCAB mortality was higher with respect to other studies; however, we included high-intermediate risk patients with low LVEF; moreover, the only death we had was due to pneumonia in a patient with not diagnosed emphysema and chronic obstructive pulmonary disease, stressing the importance of preoperative CT to assess chest abnormalities and any lung disease. Importantly, this study includes the first 20 consecutive RA-MIDCAB patients performed at our institution. We had no intraprocedural conversion to full-sternotomy, need for rescue cardiopulmonary bypass or major complication suggesting that despite being in the early learing curve, this strategy did not provide inferior results to standardized long-time performed OPCAB in intermediate/high-risk patients with impaired LV function. Algoet’s also reported fewer RBC transfusions and shorter ICU admission time in the RA-MIDCAB group. Our study confirms these findings; indeed, patients in the RA-MIDCAB group also had significantly shorter ICU and total hospital stays. Similar findings, showing shorter hospital stay, were also observed in patients who underwent robotic-guided PCI as demonstrated by Viscusi et al. [30] with non-inferior outcomes at last follow-up suggesting that robotic-assisted procedure may improve patients’ outcomes. Bay et al. also showed that at 1-year patients treated with robotic PCI for simple lesions experienced comparable outcomes with respect to those treated manually supporting this hypothesis. In our series, the incidence of RBC transfusion was only slightly lower in the RA-MIDCAB group and did not reach a statistical significance. However, the only patient in the RA-MIDCAB group who required more than 1 RBC was on DAPT until the day before surgery, leading to an increased bleeding risk. The occurrence of POAF also tended to be lower in the RA-MIDCAB group (*p* = 0.06). Despite not being significant in our study, similar findings were reported by other authors (19, 24, 28), suggesting that less manipulation of the heart during RA-MIDCAB with a smaller pericardiotomy may result in weaker postoperative pericardial inflammation. The incidence of POAF in patients with low LVEF [27] reported in the literature was lower, likely because of differences in postoperative management of perioperative risk factors. Nevertheless, our higher rate of POAF did not lead to an increased incidence of strokes during follow-up. Furthermore, patients who underwent RA-MIDCAB showed lower postoperative troponin values, possibly because the number of patients with LAD CTO was higher than in the OPCAB group. The high number of patients with LAD CTO might also have contributed to the less evident improvement in LVEF observed in the RA-MIDCAB group; however, patients in this group had a shorter follow-up time, which might have prevented the observation of the effect of hibernated myocardium retrieval.

Importantly, our study demonstrates for the first time that RA-MIDCAB is not inferior in terms of freedom from mortality, repeat revascularization, and stroke compared to OPCAB in high/intermediate-risk patients with moderately reduced LVEF. This finding was maintained after adjustment for major confounders, including age, sex, and the number of previous MI and PCI procedures, which were significantly higher in the RA-MIDCAB group.

Use of HCR or reverse-HCR in the RA-MIDCAB patients was more common, leading to complete revascularization of all lesions, and was not systemically performed in all OPCAB patients, however the difference in rate of complete revascularization between the two groups was not significant in our study. Moreover, after revision of all uncomplete cases, we found no revascularization of functionally significant vessels in only one of the OPCAB patients, while in the remaining three cases the vessels were deemed not amenable for further procedures. Other groups demonstrated the efficacy of HCR and reverse HCR using RA-MIDCAB, showing excellent mid-term survival and freedom from repeat revascularization (27,29,30). Our freedom from events in the RA-MIDCAB group was similar to the one reported by Peev et al., who analyzed patients with depressed LVEF treated with TECAB [27]. At 3 years, they reported a mortality of 27%; however, the cardiac-related mortality was set at 5%, as in our experience.

The major limitations of the study are: 1) The small sample size that might have reduced the power of statistical analysis limiting further statistical analysis as propensity matching, sensitivity analyses excluding cases who underwent PCI and more refined adjustment strategies that might have resulted in underpowered results. With such a small number of treated patients, estimated treatment probabilities may be unstable and highly influenced by individual observations. Second, the two groups showed clinically relevant imbalances in previous myocardial infarction and previous PCI. In a small cohort, these imbalances may lead to limited covariate overlap and extreme propensity scores. IPTW would consequently assign disproportionately large weights to a few patients, increasing variance and potentially producing unstable or misleading estimates rather than improving confounding control. 2) Adjustment for covariates that differed between the two populations was done by inserting each covariate singularly; we acknowledge that a simultaneous adjustment for multiple covariates would theoretically provide a more comprehensive control of confounding; however, because of the limited number of primary outcome events, fitting a multivariable model including all baseline imbalances would have resulted in severe overfitting and unstable estimates. 3) The difference in the length of follow-up that was longer for the OPCAB group potentially biasing statistical analysis; this bias was corrected by limiting the Cox regression analysis to one year and using the RMST analysis to emphasize any procedure-related difference in the primary outcome. 4) The retrospective nature of the study and a not standardized follow-up protocol that was based on phone calls and clinical charts revision; despite this the two populations were similar in terms of most baseline characteristics not affecting further comparison. In particular there was a higher number of hybrid and reverse hybrid revascularization leading to complete revascularization in the RA-MIDCAB group, but after charts revision of four patients not fully revascularized in the OPCAB group only one presented a functionally viable vessel while all other presented coronary artery branches deemed not suitable for PCI because of dimension and/or distribution.

## 5. Conclusions

RA-MIDCAB is not inferior to conventional off-pump CABG in terms of survival, repeat revascularization, and occurrence of stroke in intermediate/high-risk patients with reduced LVEF. Patients treated with RA-MIDCAB showed a 36-day longer freedom from events time at 1 year after surgery. Despite the limitations, deriving from a small study population and the paucity of adverse events, our explorstory findings suggest that patients at intermediate/high-risk patients with reduced LVEF might benefit from RA-MIDCAB instead of conventional surgery. Studies including larger populations and with longer follow-up will possibly confirm our preliminary findings.

## Figures and Tables

**Table 1:** Baseline and demographic characteristics.

	MIDCAB	OPCAB	
	Mean (SD), Median (I–III Quartile), N (%) Pts 20	Mean (SD), Median (I–III Quartile), N (%) Pts 52	*p* Value
Age (years)	69 ± 10	73 ± 9	0.092
Male	18 (90)	41 (79)	0.330
BMI (Kg/m^2^)	25 ± 3.6	27 ± 4.9	0.193
EuroSCORE	3.8 ± 1.9	3.5 ± 2.4	0.384
STS Score	3 ± 2	2.4 ± 2	0.577
Chronic kidney disease	3 (15)	9 (17.3)	0.682
Hemodialysis	1 (5)	1 (1.9)	0.481
eGFR (mL/min/1.73 m^2^)	60 ± 27	64 ± 22	0.325
Systemic arterial Hypertension	15 (75)	48 (92)	0.140
Diabetes on Insulin therapy	6 (30)	19 (36)	0.783
COPD	3 (15)	4 (7.7)	0.338
Previous myocardial infarction	9 (45)	6 (11.5)	0.003
Previous PCI	8 (40)	7 (13.4)	0.022
Previous stroke	0	5 (9.6)	0.313
Atrial fibrillation/flutter	4 (20)	4 (7.7)	0.206
Pace-maker	1 (5)	5 (9.6)	1.000
ICD	1 (5)	1 (1.9)	0.481
NYHA class II III IV	0 19 (95) 1 (5)	1 (1.9) 49 (94.2) 2 (3.9)	0.806
Previous hospitalization for heart failure	1 (5)	4 (7.7)	1.000
Beta-blocker therapy	10 (50)	39 (75)	0.052
ACE-i therapy	8 (40)	18 (34.6)	0.785
ARB therapy	1 (5)	11 (21.1)	0.159
ARNI therapy	3 (15)	4 (7.7)	0.388
MRA therapy	6 (30)	13 (25)	0.767
SGLT-2-i therapy	3 (15)	12 (23.1)	0.534
Diuretic therapy	8 (40)	30 (58)	0.199
DAPT	1 (5)	1 (1.9)	0.481
Preoperative Levosimendan	0	2 (3.9)	1.000

MIDCAB: Minimally Invasive Direct Coronary Artery Bypass; OPCAB: Off-Pump Coronary Artery Bypass; BMI: Body Mass Index; COPD: chronic obstructive pulmonary disease; PCI: percutaneous coronary intervention; ICD: Implantable Cardioverter Defibrillator; ACE: Angiotensin Converting Enzyme; ARB: Angiotensin Receptor Blocker; ARNI: Angiotensin Receptor-Neprilysin Inhibitor; MRA: Mineracorticoid Receptor Antagonists; SGLT-2-i: Sodium-Glucose Cotransporter-2 inhibitors; DAPT: dual antiplatelet therapy.

**Table 2:** Baseline-echocardiographic parameters.

	MIDCAB	OPCAB	
	Mean (SD), Median (I–III Quartile), N (%) Pts 20	Mean (SD), Median (I–III Quartile), N (%) Pts 52	*p* Value
LVEF (%)	40 ± 13	45 ± 9	0.454
LVEDD (mm)	55 ± 8	56 ± 7	0.653
IVSd (mm)	12 ± 1	11 ± 2	0.877
PWd (mm)	11 ± 1	10 ± 1	0.434
Moderate/Severe MR	6 (30)	16 (31)	1.000
RV dysfunction No Mild Severe	16 (80) 3 (15) 1 (5)	47 (90) 5 (10) 0	0.125
Moderate pulmonary hypertension	2 (10)	1 (1.9)	0.194
TR grade No Mild Moderate	8 (40) 12 (60) 0	25 (48.1) 26 (50) 1 (1.9)	0.576

MIDCAB: Minimally Invasive Direct Coronary Artery Bypass; OPCAB: Off-Pump Coronary Artery Bypass; LVEF: left ventricular ejection fraction; LVEDD: Left Ventricular End Diastolic Diameter; IVSd: interventricular septum thickness in end-diastole; PWd: Posterior Wall thickness in end-diastole; MR: Mitral Regurgitation; RV: Right Ventricle; TR: Tricuspid Regurgitation.

**Table 3:** Perioperative results.

	MIDCAB	OPCAB	
	Mean (SD), Median (I–III Quartile), N (%) Pts 20	Mean (SD), Median (I–III Quartile), N (%) Pts 52	*p* Value
Skin-to-skin time (minutes)	196 ± 66	182 ± 66	0.048
Complete revascularization	20 (100)	48 (92)	0.202
New onset POAF	4 (25)	16 (31)	0.06
Sepsis	0	3 (6)	0.268
Wound dehiscence	0	1 (2)	0.528
Intraprocedural Inotropic support	4 (20)	6 (11.5)	0.352
IABP	0	1(2)	0.532
Peak troponin (mcg/L)	133.4 ± 92.3	524.6 ± 93.4	<0.01
RBC Transfusion > 1 unit	1(5)	11(21)	0.099
OT intubation > 6 h	0	24 (46)	0.001
ICU stay > 24 h	6 (31)	18 (35)	0.05
Total length hospital stay (days)	4.6 ± 1.5	7.4 ± 2.5	0.029
Overall mortality	0	2 (3.8)	0.374
Surgical revision for bleeding	1 (5)	1 (2)	0.486
AKI	0	2 (3.8)	0.374
Discharge to rehabilitation	8 (40)	35 (70)	0.045
Diuretic therapy at discharge	12 (60)	42 (84)	0.047
Peak creatinine (mg/dL)	1.4 ± 1.2	1.4 ± 0.6	0.752
LVEF at discharge (%)	50 ± 13	51 ± 9	0.824
LVEF at last follow-up (%)	50 ± 10.4	50 ± 11	0.759
∆LVEF (baseline—follow-up) (%)	5	8	0.071

MIDCAB: Minimally Invasive Direct Coronary Artery Bypass; OPCAB: Off-Pump Coronary Artery Bypass; POAF: post operative atrial fibrillation; IABP: Intra-Aortic Balloon Pump; RBC: red blood cells; OT: oro-tracheal; ICU: intensive care unit; AKI: acute kidney injury; LVEF: left ventricular ejection fraction.

## Data Availability

The data presented in this study are available on request from the corresponding author due to privacy concerns.
